# Stimulation Therapy to Induce Mothers: Protocol for a Multicenter Randomized Controlled Trial

**DOI:** 10.2196/63463

**Published:** 2024-08-29

**Authors:** Danna Tortal, Veronika Shabanova, Sarah Taylor, Xiao Xu, Molly McAdow, Bethany Stetson, Sarah McCollum, Ester Sanchez, Amie Adjakple, Jessica Leventhal, Moeun Son

**Affiliations:** 1 Yale School of Medicine New Haven, CT United States; 2 Columbia University Vagelos College of Physicians and Surgeons New York, NY United States; 3 Northwestern University Feinberg School of Medicine Chicago, IL United States; 4 Yale University New Haven, CT United States; 5 Weill Medical College of Cornell University New York, NY United States

**Keywords:** nipple stimulation, nipple stimulation therapy, breast pump, labor induction, oxytocin, lactation

## Abstract

**Background:**

More than 1 million women have their labor induced in the United States each year, and synthetic oxytocin infusion is the most common method used. However, compared to spontaneous labor, medical induction is resource intensive, has increased obstetric risks, and is associated with less successful breastfeeding. In contrast to the endogenous oxytocin hormone, which is released in a pulsatile fashion in the brain, synthetic oxytocin is continuously infused intravenously, resulting in important limitations related to efficacy, safety, and cost. Akin to spontaneous labor contractions, infant suckling of the breast nipple is known to stimulate the pulsatile release of endogenous oxytocin from the posterior pituitary gland. Nipple stimulation therapy via an electric breast pump similarly stimulates endogenous oxytocin release and may be a favorable inpatient method for patients undergoing labor induction.

**Objective:**

This study aims to examine whether inpatient nipple stimulation therapy is an efficacious labor induction method that increases the likelihood of spontaneous vaginal delivery and sustained breastfeeding and determine whether it is a cost-effective approach.

**Methods:**

This is a multicenter, pragmatic, open-label, parallel-group randomized controlled trial of nulliparous patients with singleton gestations ≥36 weeks undergoing labor induction. This trial compares inpatient nipple stimulation therapy via an electric breast pump versus immediate synthetic oxytocin infusion without nipple stimulation. This trial including 988 nulliparas will provide adequate statistical power to detect clinically meaningful differences in delivery mode and breast milk as the sole source of nutrition for newborns at hospital discharge or 72 hours after birth.

**Results:**

The project received pilot funding in 2021 and full funding in 2023. Enrollment for this study began in November 2021 at a single site, and as of May 2024, recruitment is underway at 3 study sites. It is anticipated that enrollment will be completed by late 2026.

**Conclusions:**

Successful completion of this trial will provide rigorous data to determine whether inpatient nipple stimulation therapy with an electric breast pump can improve the way we induce labor and positively impact breastfeeding success and early infant nutrition through lactation.

**Trial Registration:**

ClinicalTrials.gov NCT05079841; https://clinicaltrials.gov/study/NCT05079841

**International Registered Report Identifier (IRRID):**

DERR1-10.2196/63463

## Introduction

### Background

Of the approximately 4 million births in the United States each year, >1 million women have their labor medically induced in the hospital [1]. Synthetic oxytocin infusion is the most common method used currently to induce labor [2]. At the molecular level, synthetic oxytocin is identical to endogenous oxytocin [3] and is therefore effective at stimulating uterine contractions [2-4]. However, synthetic oxytocin has important shortcomings. First, it is designated as a “high alert” medication [5] and requires strict oversight by the medical team for safety concerns [6-8], which is resource intensive. Second, unlike endogenous oxytocin, which is released in a pulsatile fashion, synthetic oxytocin is infused continuously, which can induce downregulation of oxytocin receptors in myometrial muscle and result in reduced efficacy [9]. Third, compared to spontaneous labor, induced labor is associated with increased risks of operative delivery, postpartum hemorrhage, and other obstetric complications, especially when oxytocin use is prolonged [10,11]. Fourth, synthetic oxytocin is infused into the peripheral circulation and minimally penetrates the blood-brain barrier [12], which precludes it from mimicking the other physiological benefits of endogenous oxytocin [13].

In contrast to synthetic oxytocin, breast nipple stimulation therapy induces a physiological process by stimulating the pulsatile release of the endogenous oxytocin hormone from the pituitary gland. Pulsatile stimulation of oxytocin [14] mimics physiological labor, which could prove nipple stimulation to be a more efficacious method of labor induction compared to continuous infusion of synthetic oxytocin. More efficient labor typically results in improved obstetric outcomes, including an increased likelihood of a spontaneous vaginal delivery. In addition, just as infant suckling of the breast nipple triggers postpartum lactation by inducing the milk ejection reflex [15], our pilot feasibility study found that nipple stimulation therapy via an electric breast pump results in early colostrum production and milk letdown during labor in many women, including nulliparas [16].

Breastfeeding has many known short- and long-term benefits, yet early discontinuation is common [17]. Breastfeeding protects against gastroenteritis, lower respiratory infection, and other infectious diseases in infancy [18-22]. Sustained breastfeeding promotes the infant’s sensory and cognitive development [23] and protects the infant from chronic diseases [20,24]. Furthermore, a history of lactation is associated with reduced maternal risks of type 2 diabetes and breast and ovarian cancers [24]. No breastfeeding or early cessation is associated with increased risks for postpartum anxiety and depression [24-26]. Given these benefits, the World Health Organization [27], the Centers for Disease Control and Prevention [28], and the American Academy of Pediatrics [29] recommend exclusive breastfeeding without any supplementary formula or water from birth until 6 months of age. However, based on US data from the Centers for Disease Control and Prevention, although 84% of infants started breastfeeding, 20% were supplemented with formula within 2 days of age, and only 25% were exclusively breastfed at 6 months as recommended [17]. Maternal perception of insufficient milk supply (PIMS) and infant weight loss are the most common reasons reported for early breastfeeding cessation [30-32]. While almost all newborns lose weight after birth due to diuresis and low enteral intake [33], it is more pronounced in those who exclusively breastfeed [33-36]. The trajectory of newborn weight loss becomes the basis for multiple clinical decisions, including the timing of hospital discharge, the need for lactation support or supplementation, and the timing and type of newborn follow-up [37,38]. A poor weight trajectory can be especially detrimental to maternal confidence [39,40], and low breastfeeding confidence in the first week post partum predicts early cessation [41,42].

Therefore, this multicenter clinical trial aims to test the central hypothesis that intrapartum nipple stimulation therapy via an electric breast pump will positively alter childbirth and early postpartum experience by increasing the likelihood of spontaneous vaginal delivery and exclusive and sustained postpartum lactation. Furthermore, studies have shown that strategies that increase the likelihood of vaginal delivery can have significant impact on public health and cost [43]. In addition, increasing breastfeeding rates would reduce reliance on infant formula and potentially reduce health care use during the early newborn period. The potential economic and public health impacts of our intervention on the success of breastfeeding and subsequent maternal and infant health are also substantial [44,45], and therefore, we also aim to examine the cost-effectiveness of this intervention.

### Aims and Hypotheses

The Stimulation Therapy to Induce Mothers (STIM) trial was designed to pursue 3 aims. First, we aim to compare the effect of inpatient nipple stimulation therapy via an electric breast pump versus synthetic oxytocin infusion without nipple stimulation (comparator) on the delivery method. We hypothesize that those randomly assigned to nipple stimulation therapy will be more likely to achieve spontaneous vaginal delivery and have fewer labor-related complications compared to those in the comparator group. Second, we aim to compare the effect of inpatient nipple stimulation therapy via an electric breast pump versus synthetic oxytocin infusion without nipple stimulation (comparator) on postpartum lactation. We hypothesize that those randomly assigned to nipple stimulation therapy during labor will be more likely to use breastfeeding as the sole source of nutrition (BSSN) at hospital discharge, which will be associated with improved maternal perception of milk supply, less severe newborn weight loss, and sustained breastfeeding for the recommended 6 months. Third, we aim to examine the cost-effectiveness of inpatient nipple stimulation therapy via an electric breast pump in comparison to synthetic oxytocin infusion without nipple stimulation (comparator). By examining the cost of care and health-related quality of life, we hypothesize that administering nipple stimulation therapy during labor will be more cost-effective than the comparator after considering their overall impact on labor and delivery, breastfeeding, and early infant nutrition and care in the first 6 months.

## Methods

### Overview and Study Design

STIM is a multicenter, pragmatic, open-label, parallel-group randomized controlled trial of nulliparas to compare the effectiveness of inpatient nipple stimulation therapy with or without adjunctive synthetic oxytocin (intervention) versus immediate synthetic oxytocin infusion without nipple stimulation (comparator) during labor induction. Randomly allocating participants to different interventions minimizes selection bias and results in groups that are comparable with regard to important confounding variables, both measured and unmeasured. A pragmatic trial design was selected so that study findings, if positive, can be directly applied and disseminated quickly and widely in the “real world.” We will follow the CONSORT (Consolidated Standards of Reporting Trials) guidelines wherever appropriate in the conduct and reporting of this trial [46]. The use of broad eligibility criteria, the simplicity and low cost of the study intervention, and the multicenter design with patient diversity increase generalizability and promote direct application of the findings.

### Ethical Considerations

This study is approved by the Yale University Institutional Review Board (IRB), which serves as the single IRB (approval 2000031338). The study is approved by the Weill Cornell Medicine IRB (23-05026095) and Northwestern University IRB (STU00219048) with reliance agreements to the Yale University IRB. The study was registered in Clinicaltrials.gov (NCT05079841) before the initiation of the study. All study participants are required to sign a written informed consent form in English or Spanish to participate in study activities before their enrollment. All participants, regardless of study group, are compensated up to $50 USD ($10 USD at each of the 5 survey study points) for completion of study surveys. In addition, all participants randomized to the nipple stimulation therapy group receive a hands-free pumping bra (valued at $30 USD) and all participants randomized to the comparator group receive a pack of diapers (also valued at $30 USD). the patient study data are all collected by trained certified staff on REDCap, a secure data software. All data will be de-identified at the analysis phase.

### Study Setting and Population

There are 3 planned recruiting sites for the STIM trial: Yale New Haven Hospital (YNHH) in New Haven, Connecticut; the Alexandra Cohen Hospital for Women and Newborns at NewYork-Presbyterian Hospital (NYP)—Weill Cornell Medicine in New York, New York; and the Prentice Women’s Hospital at Northwestern Memorial Hospital (NMH) in Chicago, Illinois.

Eligible participants are identified among patients admitted to the labor and delivery units for planned delivery at each recruiting site. We are using broad inclusion criteria to ensure the generalizability of our results. In brief, eligible patients are nulliparas with singleton gestations at ≥36 weeks 0 days, aged ≥18 years, and who are planned to undergo labor induction with synthetic oxytocin. Exclusion criteria are limited to conditions for which nipple stimulation, synthetic oxytocin, or labor attempt are contraindicated or the fetus is thought to be at higher risk for admission to the neonatal intensive care unit after birth. More specific eligibility criteria are listed in Textbox 1.

**Eligibility criteria of the Stimulation Therapy to Induce Mothers trial.**

**Inclusion criteria**

- Age ≥18 years
- Nulliparous
- Live singleton gestation ≥36 weeks
- Intact or ruptured membranes (if amniotic membranes ruptured, <3 contractions per 10-minute period, averaged over 30 minutes regardless of cervical examination)
- Able to give informed consent
- Able to understand and speak English or Spanish

**Exclusion criteria**

- Presence of tachysystole, recurrent variable or late fetal decelerations, or fetal bradycardia within 30 minutes before enrollment
- Nonvertex-presenting fetus at enrollment
- Planned for cesarean delivery or contraindication to labor by institutional policy (eg, active genital herpes infection, placenta previa, vasa previa, and history of cavity-entering myomectomy)
- HIV infection
- History of mastectomy or contraindication to nipple stimulation
- Known allergic reactions to components of the electric breast pump or to synthetic oxytocin intravenous solution
- Significantly impaired consciousness or executive function (eg, intubated or sedated)
- Fetus suspected to be at an increased risk for neonatal intensive care (eg, major fetal anomaly, which is defined as a prenatally diagnosed anomaly anticipated to require neonatal intensive care unit admission; alloimmunization; and severe fetal growth restriction, defined as prenatally suspected estimated fetal weight less than the third percentile or abnormal umbilical artery Doppler assessment showing absent or reversed flow in diastole)

### Study Interventions

#### Overview

The active arm (study intervention) in this trial is intrapartum nipple stimulation therapy via an electric breast pump (with or without adjunctive synthetic oxytocin). The control arm (current standard management) is immediate synthetic oxytocin infusion without nipple stimulation therapy as the comparator. Continuous fetal cardiotocography will be required in both treatment groups.

#### Active Arm: Nipple Stimulation Therapy via an Electric Breast Pump

Nipple stimulation therapy will be initiated in lieu of starting with synthetic oxytocin. There is no maximum length of time for which nipple stimulation can be continued if it is tolerated by the participant and her fetus. The study intervention requires a minimum of 2 hours of nipple stimulation therapy without synthetic oxytocin use to be considered valid. The 2-hour threshold was chosen because our preliminary data showed that it took a median of 69 (IQR 21-80) minutes for participants undergoing nipple stimulation therapy to have an “adequate” contraction pattern (defined as at least 3 contractions in a 10-minute period, averaged over >30 minutes) [16]. After at least 2 hours (excluding breaks) of nipple stimulation therapy, synthetic oxytocin infusion may be initiated as part of a step-up treatment strategy if desired by the participant or by their primary obstetric clinician. Synthetic oxytocin infusion can be initiated as an adjunct to continued nipple stimulation therapy or can be used as a replacement. In such cases, synthetic oxytocin will be infused according to each study site’s hospital protocol. If synthetic oxytocin infusion is initiated <2 hours (excluding breaks) of attempting nipple stimulation therapy, this will be considered a crossover. The time spent performing nipple stimulation therapy will be recorded in two ways using methods of data collection that were validated in the pilot feasibility study [16]: (1) participants or their labor support person or persons will complete a study “diary” to record start and stop times, mark unilateral or bilateral nipple stimulation, and record suction settings; and (2) per hospital protocol at all study sites, the labor nurse assesses the patient and reviews the fetal cardiotocography every 15 to 30 minutes and records the method of labor induction (ie, nipple stimulation, synthetic oxytocin, or both) as well as their interpretation of the fetal cardiotocography in the electronic medical record (EMR). In addition, the labor nurse documents the start and stop times of any labor induction agent (ie, nipple stimulation, synthetic oxytocin, etc) in the EMR as per hospital protocol.

Participants randomized to the active arm will be provided with a hospital-grade electric breast pump (Medela Symphony at YNHH and NYP and Ameda Platinum at NMH), its accessories, as well as a hands-free pumping bra and will receive a tutorial from their labor nurse. Additional support will be available from the hospital lactation consultants at all study sites. Both electric pump types use technology that mimics an infant’s natural sucking rhythm with 2 distinct phases: the stimulation phase to emulate the initial light but fast sucking to start milk flowing, followed by the expression phase that emulates slower deep suck to bring out more milk faster. Nipple stimulation will be initiated on a single breast until uterine contractions are elicited and are occurring at least every 3 minutes by patient report or based on the tocodynamometer. If stimulation of either breast does not result in the desired contraction pattern after 30 minutes, stimulation of bilateral breasts will be attempted. Therapy will be performed continuously with the pump suction set to the pressure (measured in mm Hg) most tolerated by the participant and can be self-adjusted by the participant. Similar to how synthetic oxytocin is infused and titrated, nipple stimulation therapy will be “titrated” based on the fetal cardiotocography. If there are >5 contractions in 10 minutes, the pump suction setting will be reduced or turned off until the contraction pattern is in the desired range to avoid tachysystole. If uterine contractions start to occur less than every 3 minutes by patient report or based on the tocodynamometer, the pump suction setting will be increased or turned back on (if it had been turned off) until the desired contraction pattern is obtained again. The pump can be temporarily turned off or the suction settings can be temporarily decreased per patient request even if the contraction pattern is in the desired range; however, it is not recommended for >15 minutes at a time.

#### Comparator Arm: Immediate Synthetic Oxytocin Infusion Without Nipple Stimulation Therapy

Participants randomized to the control arm will receive immediate synthetic oxytocin infusion, which is administered and dosed according to each study site’s protocol. All recruiting sites will use an oxytocin dosing protocol with an initial and incremental dose rate increase of 2 mU/min. Premixed oxytocin intravenous solutions are infused via a programmed smart pump. Dosage may be increased every 15 to 40 minutes depending on the hospital site. The oxytocin maximum infusion dose rate differs at each study site per hospital policy: 20 mU/min at YNHH and 40 mU/min at NYP and NMH. Rate changes or discontinuation of oxytocin administration are based on the assessment of the following factors: fetal status, contraction status, and maternal coping. Per protocol at all study sites, the labor nurse assesses the patient and reviews the fetal cardiotocography every 15 to 30 minutes and records the method of labor induction (ie, synthetic oxytocin) as well as their interpretation of the fetal cardiotocography in the EMR. In addition, the labor nurse documents the start and end times of any labor induction agent (ie, synthetic oxytocin) in the EMR as per hospital protocol. If nipple stimulation is performed at any time, this will be considered a crossover.

### Additional Study Procedures

For participants assigned to nipple stimulation therapy, those who express colostrum or breast milk during the stimulation process can collect and store it for postnatal feeding. Supplies for safe collection and storage are provided. All participants, regardless of group assignment, are asked to report their pain score before and 1 hour after the start of the intervention using the visual analog scale (VAS) [47]. In addition, all participants, regardless of group assignment, are asked to complete electronic surveys at 5 different study timepoints: at the time of enrollment, 1 to 3 days post partum, 2 weeks post partum, 4 to 12 weeks post partum, and 6 months post partum (Table 1). Surveys are administered using the REDCap (Research Electronic Data Capture; Vanderbilt University) survey tool, and weblinks and QR codes are provided to participants via email or SMS text message (whichever the participant prefers). Paper versions and survey administration via phone call are also available if requested.

**Table 1:** Schema of participant procedures as part of enrollment and study outcomes.

Participant procedure		At study enrollment (inpatient)	Labor and birth (inpatient)	Postpartum days 1-3 (inpatient)	Postpartum week 2 (follow-up)	Postpartum weeks 4-8 (follow-up)	Postpartum month 6 (follow-up)
Baseline procedures		✓					
Sign medical release forms for participant and soon-to-be newborn		✓					
IFI^a^ survey		✓					
EQ-5D survey		✓					
**Aim 1: delivery mode**			✓				
	Intrapartum nipple stimulation diary (study intervention group only)		✓				
	Primary outcome: spontaneous vaginal delivery		✓				
	Secondary (exploratory) outcomes: time to delivery, cesarean, OVD^b^, intraamniotic infection or PP^c^ endometritis, and PPH^d^		✓				
**Aim 2: lactation**			✓	✓	✓	✓	✓
	Primary outcome: breastfeeding as the sole source of infant nutrition			✓			
	Secondary outcomes:			✓			
	Survey about intrapartum milk letdown and colostrum collection			✓			
	Maximal newborn weight loss			✓			
	PIMS^e^ survey				✓		
	MBFES^f^ survey					✓	
	Breastfeeding continuation survey						✓
**Aim 3: cost analysis**		✓	✓	✓	✓	✓	✓
	Income and education level questions	✓					
	Employment status and return to work	✓				✓	✓
	Delivery hospitalization encounter cost (participant)		✓				
	Birth hospitalization encounter cost (infant)		✓				
	EQ-5D survey			✓	✓	✓	✓
	EQ-TIPS survey			✓	✓	✓	✓
	Infant feeding method			✓	✓	✓	✓
	Breast pump use				✓	✓	✓
	Workplace absenteeism and presenteeism						✓
	Household productivity loss						✓
	Postpartum medical care after hospital discharge (participant)						✓
	Postnatal medical care after hospital discharge (infant)						✓
	Travel to and from postpartum care office (participant)						✓
	Travel to and from pediatrician’s office (infant)						✓

^a^IFI: infant feeding intention.

^b^OVD: operative vaginal delivery.

^c^PP: postpartum.

^d^PPH: postpartum hemorrhage.

^e^PIMS: perception of insufficient milk supply.

^f^MBFES: maternal breastfeeding evaluation sale.

### Recruitment, Assignment, and Allocation

All patients who are scheduled for labor induction or are admitted to the hospital’s labor and birth unit to undergo labor induction are screened for study eligibility by a trained member of the investigative team. With the assent of their primary obstetric clinician, patients meeting eligibility criteria are approached for potential recruitment. The study is explained in detail, and all questions will be answered before signing written informed consent to participate in the study. Patients can be consented before completion of cervical ripening (if cervical ripening is needed) but will not be randomized until the medical team confirms that the patient is ready to start oxytocin and therefore eligible for randomization.

At each study site, enrolled participants are randomly assigned in a 1:1 ratio to both study groups. A web-based randomization sequence was prepared by an independent statistician using blocks of variable sizes, stratified by study site and amniotic membrane status (intact vs ruptured). The advantage of this method is that it provides a good probability of balance, and future assignments are unpredictable. A participant’s group assignment is obtained only after the participant is confirmed to continue to meet inclusion criteria, and a study number is entered and locked in using REDCap. Although blinding of participants and their obstetric teams would be ideal, blinding is clearly not possible in this trial. We will minimize systematic bias by applying the same standard procedures for other labor and management strategies between groups at each study site. Furthermore, the group assignment of participants will not be considered by the study staff collecting maternal and neonatal outcomes. Importantly, the main outcomes of spontaneous vaginal delivery and exclusive breastfeeding at hospital discharge are objective measures.

### Study Outcomes

The primary outcome (aim 1) is spontaneous vaginal delivery, defined as delivery without the use of forceps, vacuum, or cesarean, as it is the most desirable obstetric outcome for laboring women. Compared to a cesarean delivery, spontaneous vaginal delivery is associated with lower maternal and neonatal morbidity, including hemorrhage, wound infection, endometritis, and prolonged hospitalization [48]. Furthermore, once a primary cesarean is performed, the risk of delivery by cesarean for future pregnancies is significantly increased [49], further escalating maternal morbidity. Although operative vaginal delivery (ie, forceps or vacuum) is associated with lower maternal morbidity compared to cesarean delivery, it carries higher risks for maternal pelvic floor and neonatal injury than spontaneous vaginal delivery [50]. Secondary outcomes (aim 1) include time to delivery among those who achieve spontaneous vaginal delivery, cesarean delivery, operative vaginal delivery, intraamniotic infection or postpartum endometritis, and postpartum hemorrhage. Labor agentry [51], birthing experience satisfaction [52,53], and depression scale [54] scores will also be examined.

The main outcome for aim 2 is BSSN at the time of postpartum discharge from the delivery hospitalization or within the first 72 hours after birth. This time point is chosen because it is most likely to be causally linked to the study intervention, avoids the risk of attrition bias, and predicts the likelihood of sustained breastfeeding for the recommended 6 months post partum [55]. BSSN is defined as the infant receiving breast milk without any supplementary formula or water within the first 72 hours of life. The infant feeding method (breast milk only, mixed feeding that includes both breast milk and infant formula, or infant formula only) is standardly documented in the EMR at least daily until the day of birth hospitalization discharge, and these data will be abstracted from the EMR by trained study staff. The baseline strength of breastfeeding intention will be assessed using a validated infant feeding intentions scale [56,57] at the time of trial enrollment. The total score of the 5-question Infant Feeding Intentions scale ranges from 0 (very strong intention not to breastfeed at all) to 16 (very strong intentions to provide breast milk as the sole source of milk for the first 6 months). Secondary outcomes (aim 2) include maximal percent newborn weight loss within the first 72 hours of life, patient-reported PIMS in early lactation, patient-reported satisfaction with breastfeeding, and the rate of sustained breastfeeding for the recommended 6 months post partum. These secondary outcomes are chosen to explore potential reasons if, as we hypothesize, there are differences seen in BSSN or sustained breastfeeding. Maximal percent newborn weight loss is defined as the difference between birth weight and the lowest weight recorded subsequently up to 72 hours of age during the birth hospitalization, calculated as a percentage of the birth weight, as is typically done daily in clinical practice. It is standard hospital practice at all study sites to regularly weigh each newborn during their birth hospitalization. Infants are weighed naked using a digital scale, and the weight is expressed in kilograms. At all study sites, newborns are weighed at birth and then at least daily until the day of discharge. The second weight measured after birth is usually performed after at least 6 hours after birth. Weighing is typically discontinued if the newborn regains his or her birth weight before discharge. Infant birth weights are recorded in a standardized fashion in specific EMR flow sheets at all study sites. The date and time of each weight are recorded, which allows the calculation of precise age at the time of weight. Patient-reported PIMS will be assessed using the validated PIMS survey [58]. The 14-question PIMS uses a Likert scale for each statement, and all responses are grouped into positive (consistent with a perception of adequate milk supply) and negative (consistent with PIMS) responses. Patient-reported satisfaction with breastfeeding will be assessed using the validated Maternal Breastfeeding Evaluation Scale (MBFES) [59,60]. The total score of the 30-question MBFES ranges from 30 to 150, with higher scores reflecting greater satisfaction, indicating a positive evaluation of the breastfeeding experience. To assess continuation of breastfeeding at 6 months post partum, participants are asked via a survey whether they are exclusively breastfeeding, mixed feeding with breast milk and formula, or exclusively formula feeding.

Outcomes assessed in aim 3 include the following: (1) cost of relevant care and (2) health-related quality of life. Both outcomes will be measured for the mother-infant pair. Cost of care will include direct medical cost, direct nonmedical cost, and indirect cost. The direct medical cost will encompass hospital facility costs and provider professional fees associated with relevant care (maternal labor and delivery hospitalization, newborn’s initial birth hospitalization, maternal postpartum care, and infant’s postnatal pediatric visits). Direct nonmedical cost will account for breast pump use at home, infant formula use, and transportation to relevant care. Indirect cost (ie, maternal productivity loss) will measure participants’ work loss in the postpartum period based on income and employment survey questions. As the project will span multiple years, we will adjust all cost estimates for inflation using the Consumer Price Index (CPI) to reflect a constant-year US dollar [61]. Health-related quality of life for participants will be measured by the EQ-5D instrument [62]. EQ-5D is a validated instrument for measuring and valuing health. It includes a descriptive system assessing 5 dimensions of health (mobility, self-care, usual activities, pain or discomfort, and anxiety or depression), along with a VAS (EQ VAS) assessing overall health ranging from worst to best imaginable health. Response to each dimension includes 5 severity levels (no, slight, moderate, severe, or extreme problems). Participants’ response to the 5 dimensions of the EQ-5D descriptive system will be converted to a utility score using a validated US population–based algorithm [63]. Utility score ranges from 0 to 1, with 0 referring to a health state equivalent to death and 1 equivalent to perfect health. EQ-5D is a recommended and widely used instrument for measuring utility scores in clinical trials and in maternal health research [64,65]. For infants, the South Africa (English) EQ-TIPS paper proxy 1 questionnaire (previously known as Toddler and Infant Health-Related Quality of Life [66]) is completed by participants (as proxy reporters for their infants). This questionnaire has been shown to be valid and reliable for use in very young children [66]. It contains a 6-dimension descriptive system (movement, play, pain, social interaction, communication, and eating) with 3 severity levels (no, some, or a lot of problems), along with a VAS assessing an infant’s overall health. As an algorithm for calculating utility scores based on the EQ-TIPS descriptive system is not available yet, we anticipate using a linearly transformed VAS score to approximate a utility score on a 0 to 1 scale.

### Data Collection and Management

Labor course and outcome data of randomized participants and their newborns are collected by study staff through direct interview and chart review of the EMR. Data are collected on standardized forms in REDCap, an established, secure, web-based capture and management tool developed at Vanderbilt University and supported by the Yale Center for Clinical Investigation, on which nearly all responses are precoded.

All participants will be assessed for aims 1 and 2 and their secondary outcomes. Maternal follow-up will occur until 6 months post partum. We expect that all participants will be included for the coprimary outcomes as all study sites provide maternity and newborn care with the highest levels designated by each respective state (Connecticut, New York, and Illinois), and early out-of-hospital transfers are not expected. Aim 3 will include all participants and newborns with complete cost data, with regular follow-up for up to 6 months.

Data on cost of care will be collected from multiple sources. First, accounting databases are available at all study sites and use advanced platforms that allow for the extraction of detailed data on hospital facility costs. Physician professional fee data will also be extracted for services provided by physicians and other practitioners. If these data are not accurately available, relevant fee schedules from the Centers for Medicare and Medicaid Services [67] will be used alternatively. Hospital facility costs and physician professional fees will be determined for the delivery hospitalization encounters of the participants and the birth hospitalization encounters of their newborns. As hospital-grade electric breast pumps are multiuser pumps, its cost for each participant will be allocated based on duration of use and the expected total life span of the pump. Second, the cost of medical care after hospital discharge will be based on a roster of outpatient visits, emergency department visits, and rehospitalizations for both participants and their newborns. Third, participants are asked to complete surveys to report personal income; education level; employment status and return to work; workplace absenteeism, presenteeism and household productivity loss; infant formula use; and breast pump use at home. Fourth, travel costs for the care of participants and their newborns will be estimated based on round trip miles between the home zip code and the zip codes of the postpartum care office and the pediatrician’s office.

### Sample Size and Power

The sample size for the trial is based on the primary outcome of spontaneous vaginal delivery for aim 1 (Table 2). All sample size and power estimates are based on 2-tailed tests. This is important because we will be powered to detect both increases and decreases in outcomes with intrapartum nipple stimulation therapy (intervention) versus immediate synthetic oxytocin infusion without nipple stimulation (comparator). The expected rate of spontaneous vaginal delivery in the setting of immediate synthetic oxytocin infusion without nipple stimulation used for the sample size estimation is based on averaged institutional data from the 3 study sites: (62%+66%+70%)/3=66%. Accounting for a 5% attrition rate, we estimate that a total of 988 participants (494 in the nipple stimulation therapy group and 494 in the immediate synthetic oxytocin infusion without nipple stimulation group) will be sufficient to detect a minimum of 9.8% absolute difference (estimated 75.8% with nipple stimulation therapy vs 66% in the comparator group) in spontaneous vaginal delivery with 90% power (2-sided α=.05). Of note, 80% statistical power would be needed to detect a minimum absolute difference of 8.6% (74.6% vs 66%).

**Table 2:** Sample size estimation for primary outcome.

Detectable absolute difference (%)	Anticipated with nipple stimulation therapy (%)	Total sample size across 2 study groups for 90% power, N
9.4	79	988
9.6	78	988
9.7	77	988
9.8	76	988
9.9	75	988
10.0	74	988
10.1	73	988
10.1	72	988

At first glance, the anticipated minimum of approximately 10% absolute increase in the spontaneous vaginal delivery rate in our proposed trial appears modest. On the contrary, because the potential public health impact is large, this effect size is significant. It is estimated that 40% of the 1 million women who undergo inpatient labor induction each year in the United States are nulliparas. Therefore, a minimum of approximately 10% increase in spontaneous vaginal delivery corresponds to 40,000 more spontaneous vaginal deliveries and 40,000 fewer operative and cesarean deliveries each year. Avoiding 40,000 operative or cesarean deliveries each year will have a substantial impact on overall morbidity and health care resource use in the United States and can have broad implications beyond the United States.

The sample size of 988 for the primary outcome will be sufficient to detect a 9.4% or 10.8% absolute difference in BSSN at the time of delivery and hospitalization postpartum discharge with 80% power and 90% power, respectively (2-sided test, α=.05). This represents the difference between an expected BSSN rate of 45.5% in the setting of synthetic oxytocin infusion without nipple stimulation (average rate at the 3 study sites based on institutional data: 44%+50%+42.5%/3=45.5%) and 54.9% (with 80% power) or 56.3% (with 90% power) with nipple stimulation therapy.

Assuming a 15% attrition rate at 2 weeks post partum (the time frame for our primary cost-effectiveness analysis in aim 3), the sample size of 988 will have 90% power for detecting a standardized effect size of 0.224 (0.194 for 80% power) for the between-group difference in cost and difference in quality-adjusted life year (QALY; 2-sided test, α=.05). This is considered a small effect size based on conventional criteria; hence, if we observe an even larger effect size, we should be sufficiently powered [68].

### Data Analysis Plan

#### Overview

Analyses will follow the intention-to-treat principle in which participants will be analyzed in the group to which they were randomized, regardless of whether they received the assigned intervention. Descriptive statistics will characterize the group of individuals recruited and investigate the comparability of the 2 study groups at baseline. Formal statistical testing will be limited to select baseline characteristics considered to be prognostic factors for the primary outcome, including hospital admission BMI, primary indication for labor induction, Bishop score at randomization, and birth weight. Categorical variables will be compared between trial groups by using the chi-square or Fisher exact tests as appropriate, and continuous variables will be compared using Student *t* tests (2-tailed) or Wilcoxon rank sum tests as appropriate. Distributions of continuous variables will be assessed by visual inspection of histograms.

The primary outcome (spontaneous vaginal delivery) and other categorical secondary outcomes will be compared between trial groups using the chi-square or Fisher exact tests as appropriate. The estimates of the relative risk and 95% CIs associated with the primary and secondary outcomes will be calculated using the Agresti and Coull method. The time to event regression analyses for labor length (regardless of delivery mode) and labor length censored for cesarean will be evaluated by Kaplan-Meier estimates and plots and tested with the log-rank test, whereas Cox proportional hazards analysis will be used in adjusted analyses accounting for Bishop scores at study entry (proportionality assumption will be checked graphically with ln[-lnSurvival] plots), and results summarized using hazard ratios and 95% CI. A sensitivity analysis will be performed using the induction method that the patient actually received (per-protocol analysis) to determine whether crossovers influenced the results. The distribution of maximal percent newborn weight loss is not expected to follow a normal distribution in the population, so we plan to use Wilcoxon rank sum tests to compare the distribution of the maximal percent weight loss between treatment groups and summarize the results as medians (25th and 75th percentiles) and bootstrapped 95% CIs. Similarly, the distributions of the PIMS survey scores and MBFES survey scores are not expected to follow normal distributions in the population, so we will use Wilcoxon rank sum tests to compare the distribution of the MBFES scores between treatment groups and summarize the results using the group medians (25th and 75th percentiles) and bootstrapped 95% CIs.

#### Adjusted Analyses

We will conduct additional analyses as needed to obtain adjusted assessments of treatment effectiveness, accounting for baseline patient characteristics (covariates). The objectives of these analyses are to estimate the influence of covariates on the outcome and to use covariates to improve the estimated difference between treatment groups. The Poisson regression model (link=log) with robust SEs stratified by the study site will be used to identify and estimate the effect of multiple prognostic factors on the probability of spontaneous vaginal delivery and other categorical outcomes, with results summarized as adjusted risk ratios. For continuous secondary outcomes such as maximal newborn weight loss and PIMS and MBFES survey scores, quantile regression, for example, modeling the 50th percentile, will be considered to adjust for prognostic factors.

#### Planned Subgroup Analyses

The following prespecified subgroup analyses will be conducted: (1) study site, (2) amniotic membrane status (intact vs ruptured) at enrollment, (3) presence versus absence of maternal diabetes (inclusive of pregestational and gestational diabetes), (4) insurance type (commercial vs public insurance), (5) maternal race (non-Hispanic Black vs non-Hispanic White vs other), and (6) obesity (obese vs nonobese). In separate models, each of these subgroup variables will be included as an additional covariate in the models for outcomes of interest, plus their interaction with the treatment variable, followed by stratified analyses (by each of the above variables) of the effect of treatment on each outcome of interest.

#### Cost-Effectiveness Analyses

Cost analyses will follow recommendations by the Second Panel on Cost-Effectiveness in Health and Medicine [69]. We will use both a societal perspective (include all costs and health effects regardless of who incurs such costs or effects) and the health care sector perspective (only include direct medical cost) to best inform decision-making. Costs will be categorized by phase or type of care (labor and delivery, newborn hospitalization, postnatal care, etc) and then aggregated to calculate the total cost for each participant-infant pair. Effectiveness of the intervention will be assessed using QALY. QALY is calculated by weighting the duration of life years in each health state by its corresponding utility score. We will use utility score at the various assessment timepoints and use an area under the curve approach to calculate QALY [70]. QALY of each participant and her infant will be summed. Our primary analysis will examine costs and health effects up to 2 weeks post partum (the period when the intervention is most influential on the outcomes and less likely to be confounded by other factors occurring post partum). To capture potential longer-term impact, we will conduct a secondary analysis extending the period to 6 months post partum. Because the analytical time frame for a given participant is <1 year, we will not discount cost or QALY [69]. In addition, although EQ-5D has been demonstrated to adequately capture differences in women’s quality of life between cesarean delivery and spontaneous vaginal delivery [65], there is some concern that the 5 dimensions of the EQ-5D descriptive system may not be very sensitive to other pertinent outcomes of labor induction [71]. Therefore, we will perform a sensitivity analysis using participants’ EQ VAS rating as a utility score (with linear transformation to a 0 to 1 scale), which presumably captures a participant’s perceive overall health regardless of dimensions.

To estimate the difference in cost and difference in effectiveness between the intervention and comparator groups, we will use a generalized linear model with log link and gamma distribution for cost (given skewness in cost data) and identity link and normal distribution for QALY. The unit of analysis will be each participant-infant pair. Explanatory variable will be an indicator for intervention (vs comparator) group. All analyses will follow the intention-to-treat principle. As randomization will be stratified by study site and amniotic membrane status, we will test if they are associated with cost or QALY and then decide whether to include them as covariates in analysis [72]. Cost-effectiveness will be evaluated by calculating the incremental cost-effectiveness ratio (ICER), defined as ΔC/ΔE where ΔC denotes the estimated difference in cost between the intervention and comparator groups and ΔE reflects the estimated difference in QALY between the 2 groups. ICER informs the additional cost associated with the intervention for each additional QALY gained. We will use nonparametric bootstrap resampling to estimate 95% CI of ICER and produce a cost-effectiveness plane and cost-effectiveness acceptability curve. In special situations where the intervention leads to significantly lower cost and higher QALY, the intervention is considered the dominating strategy. We will follow the Consolidated Health Economic Evaluation Reporting Standards (CHEERS) to report the findings [73].

## Results

The project received pilot funding in 2021 and full funding in 2023 (Multimedia Appendix 1). Enrollment for this study began at YNHH in November 2021, NYP in April 2024, and NMH in May 2024. As of June 2024, a total of 320 participants have been enrolled. We anticipate to complete enrollment by late 2026, and we expect to submit the initial results for publication by 2027.

## Discussion

### Anticipated Findings

Our central hypothesis is that intrapartum nipple stimulation therapy via an electric breast pump will positively alter the childbirth and early postpartum experience by increasing the likelihood of spontaneous vaginal delivery and exclusive and sustained lactation. This hypothesis is based on the synthesis of our prior work [16] and others’ published work [74-79].

### Comparison to Prior Work

Prior studies have shown that nipple stimulation therapy induces uterine contractions [74-78] and increases endogenous oxytocin levels [79], supporting our hypothesis that it will prove to be an efficient labor induction method. Furthermore, nipple stimulation has the added benefit of inducing the milk ejection reflex [80,81]. Our prior work [16] showed that nipple stimulation therapy with an electric breast pump is feasible to perform during labor, that patients are interested in trying this induction method and protocol adherence is high, and that most patients had early colostrum production and milk letdown during labor. By studying, refining, and operationalizing the study intervention through our preliminary work [16], we are well positioned to successfully conduct an adequately powered large clinical trial.

### Strengths and Limitations

With our robust and rigorous experimental design, this multicenter clinical trial will amplify and expand on prior research in several important ways. First, this trial is testing inpatient nipple stimulation therapy in a well-defined study population at multiple study sites, which will optimize external validity and promote generalizability. Prior randomized studies included both nulliparas and multiparas [76,82] or did not consider parity [77]. This is a severe limitation given well-known differences in labor patterns, childbirth outcomes, and familiarity with breastfeeding based on parity. Our feasibility study [16] piloted the intervention in both groups and found that nulliparas were similarly willing and able to perform nipple stimulation therapy during labor. Therefore, nulliparas were chosen as the population of interest for this study because they are at greater risk for failed labor induction and early breastfeeding discontinuation compared to multiparas and, therefore, anticipated to reap the most potential benefit from the study aims. This trial also seeks to overcome the limited validity of prior studies in which eligibility criteria were poorly defined [77] or too restrictive [82]. We will use eligibility criteria that are appropriately inclusive to maximize study recruitment and enhance generalizability but are also well defined to minimize confounding factors and safeguard against harm.

Second, this trial is adequately powered to assess for clinically significant differences in 2 key outcomes. Three prior published randomized studies [76,77,82] comparing nipple stimulation versus synthetic oxytocin infusion were severely limited by small sample sizes (62 to 92 participants), and none of them examined postpartum breastfeeding. The planned sample size of 988 women will be adequately powered to detect clinically important differences in spontaneous vaginal delivery, a desirable labor outcome, and the rate of infants receiving breast milk as their sole source of nutrition at hospital discharge, a desirable postpartum outcome.

Third, this trial investigates whether inpatient nipple stimulation therapy improves lactation outcomes and comprehensively explores potential reasons such as maternal perception of milk supply and severity of early newborn weight loss. One randomized trial [83] examined nipple stimulation via breast pump versus synthetic oxytocin in the third stage of labor (ie, after birth of the newborn but before delivery of the placenta). They found that women who performed nipple stimulation were more likely to initiate breastfeeding in the first 24 hours after birth but did not examine whether breastfeeding was sustained past the first 24 hours. Alternatively, antenatal breast milk expression before labor has also been studied as a method to promote breastfeeding and prevent complications such as hypoglycemia for newborns at increased risk (eg, those born to diabetic mothers) [80]. However, the association between antenatal breast milk expression and endogenous oxytocin release has raised concerns about the possible induction of premature labor. If true, a strategy that induces breast milk expression hours before birth, like ours, may prove to be the ideal intervention.

Fourth, we will investigate the potential economic implications of this innovative study intervention. With the large number of births each year, identifying cost-effective obstetric interventions can have substantial public health and financial benefits. Inpatient nipple stimulation therapy is expected to be of low cost. Our rigorous collection and assessments of cost data along with participants’ and infants’ health-related quality of life will contribute important information to inform the cost-effectiveness of this novel intervention.

There are potential limitations that we continue to work to overcome. First, as with any prospective study, recruitment can be a challenge due to clinical volume fluctuation or low consent rates. With the recent addition of 2 recruiting sites (NYP and NMH) and the addition of more research staff to recruit during off-hours, we expect to readily achieve our planned sample size. Second, there is risk of crossover for participants in the study intervention group if synthetic oxytocin is initiated before completing 2 hours of nipple stimulation. In our feasibility study [16], the mean duration of nipple stimulation therapy was 208 (SD 28) minutes, or 3.5 hours, and attrition rate in the nipple stimulation group was only 5%, which is already captured in our sample size calculation. In addition, sensitivity analysis will be performed to address potential crossover. Participants in the intervention group can receive synthetic oxytocin as adjunctive therapy after 2 hours of nipple stimulation therapy without being considered a crossover. Even if synthetic oxytocin is used adjunctively to nipple stimulation, this is not considered a failure of treatment because these 2 treatments likely work synergistically, and intrapartum nipple stimulation can still have other benefits aside from uterine contractions. Furthermore, this is not likely to increase type I error, but in fact, it can skew our inference toward the null hypothesis or to the superiority of synthetic oxytocin infusion without nipple stimulation (comparator) because attempting nipple stimulation therapy would have only prolonged or complicated the labor induction process in cases in which it was unsuccessful. In a sensitivity analysis, we will obtain risk ratios by further recategorizing our intervention by accounting the time to initiation of synthetic oxytocin as adjunctive therapy after at least 2 hours of nipple stimulation therapy. Third, nipple stimulation therapy is controlled by the participant, which will likely prove to be a major benefit of the therapy. However, measurement of the therapy relies somewhat on patient participation as they are asked to complete a standardized diary to track what they are doing. In our feasibility study [16], the diary completion rate was >90%. In addition, per hospital protocol at all study sites, the labor nurses document the start and end times of any labor induction agent in the EMR. Therefore, measurement of adherence to study intervention protocol is expected to be reliable. Fourth, survey completion rates in our feasibility study [16] were high; however, a higher than anticipated loss to follow-up is possible. While this will not impact our primary outcomes, incomplete data would affect secondary outcomes. Multiple strategies are in place, such as reminder SMS text messages and follow-up phone calls to participants, to minimize missing data.

### Conclusions

The expected outcome of this clinical trial is that nipple stimulation therapy with an electric breast pump during labor will increase the likelihood of spontaneous vaginal birth and improve breastfeeding success and early newborn hydration and nutrition, thereby decreasing health care costs. With >1 million US women having their labor medically induced every year, successful completion of this randomized trial will help address the need to improve induction methods and may offer an opportunity to positively impact the childbirth and postpartum experience.

## Appendix

Multimedia Appendix 1 Peer review report from the Pregnancy and Neonatology Study Section - Endocrinology, Metabolism, Nutrition and Reproductive Sciences Integrated Review Group - Center for Scientific Review.

## Abbreviations

BSSN: breastfeeding as the sole source of nutrition
CHEERS: Consolidated Health Economic Evaluation Reporting Standards
CONSORT: Consolidated Standards of Reporting Trials
EMR: electronic medical record
ICER: incremental cost-effectiveness ratio
IRB: institutional review board
MBFES: Maternal Breastfeeding Evaluation Scale
NMH: Northwestern Memorial Hospital
NYP: NewYork-Presbyterian Hospital
PIMS: perception of insufficient milk supply
QALY: quality-adjusted life year
REDCap: Research Electronic Data Capture
STIM: Stimulation Therapy to Induce Mothers
VAS: visual analog scale
YNHH: Yale New Haven Hospital

## Data Availability

The data sets generated during and analyzed during this study are available from the corresponding author on reasonable request. We are committed to the open and timely dissemination of all research outcomes through the development of repositories and databases, posting data on open websites, presentations, and publications as soon as feasible after peer review. Research data will be made publicly available through open websites, presentations, and peer-reviewed publications, with any embargo periods not exceeding 1 year. We will document our methods and sources for developing and modifying any questionnaires used for the trial that may enhance data sharing in the future. In the process of data sharing, several approaches may be considered to protect privacy of the data, including release of only part of the data, altering the data in ways that will not compromise analyses, requiring outside researchers to adhere to strict confidentiality requirements, or providing access to the data through a controlled data enclave. We will assure the confidentiality of all human participants’ data and will adhere to all Health Insurance Portability and Accountability Act rules by deidentifying data as appropriate to ensure compliance with human participant confidentiality requirements. To ensure patient protection, the following procedures will be established to deidentify the data before research data sets are released: patient ID (the unique identifier for each patient consisting of a computer assigned number) will be removed, variables with low frequencies for some values that might be used to identify individual participants will be recoded and grouped into categories (eg, maternal age >40 years), and racial or ethnic groups will be collapsed if there are few individuals in certain groups or cells. The data will be evaluated to ensure the risk of reidentification is very small. We will ensure that the policies for use of the released database are followed. Before releasing the data set, an authorized individual from the institution must sign a data use agreement requiring that the database is for the use of the investigators only and cannot be copied or distributed, participant confidentiality must be maintained by not seeking or facilitating mechanisms leading to identification of individual participants, and the research investigator must show evidence of IRB or human participants review committee approval.
