# Supplementary material for: Stimulation Therapy to Induce Mothers: Protocol for a Multicenter Randomized Controlled Trial
Source: JMIR Res Protoc. 2024 Aug 29;13:e63463. doi: 10.2196/63463 (PMC11393510; doi:10.2196/63463)
Supplement: Multimedia Appendix 1 [file resprot_v13i1e63463_app1.pdf]

1R01HD111633-01 Son, Moeun

EARLY STAGE INVESTIGATOR
NEW INVESTIGATOR

RESUME AND SUMMARY OF DISCUSSION: The purpose of this application is to test the relative effectiveness of oxytocin versus breast nipple stimulation for induction of labor and postpartum lactation. Developing electric breast pump induction strategies that reduces cesarean delivery rates and promotes breastfeeding would be highly significant as it would improve health outcomes and reduce costs. The benefits to low resource countries would be particularly significant. Additional strengths of the application are the outstanding PI with demonstrated experience managing clinical trials, the complimentary expertise of co-Investigators in infant nutrition, biostatistics and cost-effective analysis, the multicenter randomized clinical trail study design that will provide meaningful guidance regardless of outcome, the preliminary data that demonstrated acceptability and provided effect size estimates and the innovation of having both delivery method and breastfeeding adherence as primary outcomes. The inclusion of a cost-effectiveness study in Aim 3 is a noted strength. Only minor weaknesses were noted and included concerns with inclusion of women who can't or won't breastfeed and the relative lack of experience managing clinical trials of the PI of the second site. Following the discussion, these concerns did not temper enthusiasm for the outstanding application and led the panel to conclude it would have a major impact on the field of labor management.

DESCRIPTION (provided by applicant): Over 1 million women have their labor induced in the United States each year, and synthetic oxytocin infusion is the most common method used. However, compared to spontaneous labor, medical induction is resource- intensive, has increased obstetric risks, and is associated with less successful breastfeeding. In contrast to endogenous oxytocin hormone which is released in a pulsatile fashion in the brain, synthetic oxytocin is continuously infused intravenously, resulting in important limitations related to efficacy, safety, and cost. Akin to spontaneous labor contractions, infant suckling of the breast nipple is known to stimulate the pulsatile release of endogenous oxytocin from the posterior pituitary gland. Nipple stimulation therapy via electric breast pump similarly stimulates endogenous oxytocin release, and our preliminary work shows that it is a feasible and acceptable inpatient method that results in a desirable uterine contraction patter n in nulliparas. Our pilot study of 100 randomized nulliparas showed that intrapartum nipple stimulation therapy decreases labor duration and trends toward a significant increase in the rate of spontaneous vaginal delivery compared to synthetic oxytocin infusion. Further, nipple stimulation reduced the dose and duration of synthetic oxytocin even when adjunctive synthetic oxytocin was used. Therefore, nipple stimulation therapy will likely prove to be an efficacious labor induction method that increases the likelihood of spontaneous vaginal delivery, and also have added physiologic benefits. For example, nipple stimulation triggers lactation by inducing the milk ejection reflex, and our preliminary work also shows that nipple stimulation therapy via electric breast pump results in early colostrum production and milk letdown in the majority of women, including first-time mothers. Earlier lactation would alleviate the most common reasons for early breastfeeding discontinuation by improving maternal perception of insufficient milk supply and the severity of weight loss that occurs in infants in the first few days of life as they establish feeding. This in turn would improve the likelihood of sustained breastfeeding for the recommended 6 months, which also has many short- and long-term benefits. Consequently, nipple stimulation therapy during labor has tremendous potential public health and cost benefits, and its success would be particularly important in areas of poverty, including developing countries. We propose a multicenter randomized trial at Yale and Northwestern Universities to compare inpatient nipple stimulation therapy via electric breast pump versus immediate synthetic oxytocin infusion without nipple stimulation for nulliparous women undergoing labor induction. This trial of 988 nulliparous women will provide adequate statistical significance to detect clinically meaningful differences in

delivery mode and breastmilk as the sole source of nutrition for newborns. Successful completion of this proposal will provide rigorous data to help us show how this novel and potentially cost-effective method can radically change the way we induce labor and positively impact breastfeeding success and early infant nutrition through lactation.

PUBLIC HEALTH RELEVANCE: Synthetic oxytocin infusion, the current standard clinical method used to induce labor, has important shortcomings. Breast nipple stimulation triggers the pulsatile release of endogenous oxytocin hormone in the brain to induce spontaneous contractions, stimulates lactogenesis, and has other physiological benefits. Therefore, the current proposal seeks to examine whether inpatient nipple stimulation therapy via electric breast pump during labor induction results in a greater likelihood of spontaneous vaginal delivery, improves the frequency of exclusive and sustained lactation postpartum, and is more cost-effective than synthetic oxytocin infusion without nipple stimulation among nulliparas.

CRITIQUE 1

Significance: 2
Investigator(s): 3
Innovation: 1
Approach: 3
Environment: 1

Overall Impact: The investigators propose an open-label, randomized controlled trail of nipple stimulation using electric breast pumps versus standard of care (continuous oxytocin infusion), hypothesizing that the pulsatile release of endogenous oxytocin stimulated by the breast pump will increase spontaneous vaginal delivery, improve breastfeeding, and decrease neonatal weight loss. The cost effectiveness of this approach will be evaluated as the third Aim. This is an important trial; labor induction is a common procedure. Additionally, promoting a labor induction strategy that enhances breastfeeding is an innovative approach and could solve an important public health issue with long-lasting impacts while also decreasing cesarean delivery. There are several minor points to the strategy that slightly dampen enthusiasm for the proposal.

1. Significance:

Strengths

- This is an important public health problem that will be adequately addressed through the study design.
- The intervention proposed will be easily implemented throughout the country and as the investigators point out in low-income countries as well.

Weaknesses

- Minor weakness: Not studying some relevant secondary outcomes such as tachysystole, fetal decelerations, and neonatal acidemia that may be relevant and reduced by stimulation inductions (and subsequently promote this as a potential outpatient intervention).

2. Investigator(s):

Strengths

- The PI is an early stage investigator who has successfully completed a single-site double-blind randomized controlled trial of >1,000 women under the mentorship of experienced investigators. She additionally successfully and independently completed a pilot randomized trial to provide data for the proposed study. Her prior experience demonstrates the skills required to lead this study
- Dr. Xu appears well qualified to lead the healthcare costs analysis.
- Dr. Taylor is an experienced investigator who brings needed neonatal expertise to the research.
- Dr. Shabanova provides critical biostatistical support (although there is an in the budget justification section that is not completed for her)

Weaknesses

- The site PI at NWM is relatively inexperienced; her only prior listed clinical trial experience is as co-investigator with Dr. Son on the oxytocin study. Although surrounded by an experienced clinical research team at NWM, given the rate of recruitment needed a more seasoned clinical investigator may be needed at NWM.

3. Innovation:

Strengths

- Formulating an induction strategy that both reduces cesarean and enhances breastfeeding is a novel concept. While nipple stimulation has been in long-standing use to induce contractions, the use of an electric or mechanical breast pump to induce labor while promoting breastfeeding has not been systematically studied.

Weaknesses

- None noted.

4. Approach:

Strengths

- Open label randomized control trial. Although never explicitly stated, a double blinded randomized trial would not be feasible with this intervention.
- Intervention is readily available and easy to implement.
- The study is well-powered to detect a clinically meaningful difference on the impact of stimulation on induction in nulliparous women.
- Nulliparous women only is an appropriate study design decision (as opposed to including multiparous women additionally).

Weaknesses

- A primary outcome of the study is exclusive breastfeeding; although intent to breastfeed is assessed at study entry, it seems that the PI would want to exclude women who do not wish to breastfeed (in particular as they may suffer more from engorgement related to nipple stimulation).
- The choice/definition of the primary outcomes is questionable. 1) Spontaneous vaginal delivery versus any vaginal delivery. Agree that SVD is preferable to OVD, but would method of induction really impact the SVD versus OVD rate? This reviewer worries that being so specific as to SVD may lose the benefit of improving vaginal delivery overall. 2) “Breastfeeding as the

sole source of nutrition at the time of delivery hospitalization postpartum discharge within the first 72 hours" – this leads to assessment of breastfeeding as the sole source of nutrition at varying time points – 24, 36, 38, 72 hours, and may not capture all cesarean discharges (some of whom may discharge at 96 hours). Additionally, it is unclear if a baby has to stay in the hospital for longer, will breastfeeding at discharge be assessed at 72 hours? It is best to pick a defined time point (preferably a clinically meaningful one that reflects continued breastfeeding later) and assess that time point, regardless of whether or not that means that the patient has to be contacted after discharge to obtain the information.

- All participants will be contacted by e-mail to complete follow up surveys. This may exclude patients that do not have e-mail from participating. Will email availability be confirmed as inclusion criteria? Consider using text messages instead of e-mail as this may increase the patient population available to participate and enhance generalizability.
- Oxytocin infusion (initiated after 2 hours of stim therapy for intervention arm and in the control arm) will be per hospital protocol; however, the hospital protocols are different (different maximum rates) and NMH allows for high-dose oxytocin. Consider not allowing high-dose oxytocin in the study and standardizing maximum rates – while the investigators large RCT did not demonstrate an impact on cesarean rates there were differences in several secondary outcomes of importance (and remaining questions that high-dose oxytocin may decrease cesarean based on other studies).
- The inclusion criteria require a Bishop score >=5. This is completely appropriate (as cervical ripening prior to oxytocin increases vaginal delivery). However, there is no description of cervical ripening agents prior to enrollment / consent – can patients participate after cervical ripening? (If no, this really limits the generalizability of the study as few nulliparous patients will present with Bishop score >=5). If yes, more details are needed regarding enrollment/management of induction until Bishop score>=5. Will there be anticipated differences between groups that should be considered? Is it anticipated that there will be a difference if somebody receives misoprostol versus cervical foley (and if cervical foley will OT use prior to study entry be allowed)? If patients are approached prior to initiation of induction, what will happen if they no longer require oxytocin after cervical ripening (e.g., the rare patient that contracts spontaneously after misoprostol placement)

5. Environment:

Strengths

- Yale and Northwestern are both academic institutions well suited to obstetric studies with long track records of successful recruitment.

Weaknesses

- None noted.

Study Timeline:

Strengths

- Appropriate time for study start up, recruitment, and analysis.

Weaknesses

- None noted.

Protections for Human Subjects:

Acceptable Risks and/or Adequate Protections

Data and Safety Monitoring Plan (Applicable for Clinical Trials Only):

Acceptable.

Inclusion Plans:

- Sex/Gender: Distribution justified scientifically
- Race/Ethnicity: Distribution justified scientifically
- For NIH-Defined Phase III trials, Plans for valid design and analysis: Not applicable
- Inclusion/Exclusion Based on Age: Distribution not justified scientifically
- Exclusion of <18 is not scientifically justified. These patients may equally benefit from stimulation therapy and increase the chance of breastfeeding success in this high-risk population.

Vertebrate Animals:

Not Applicable (No Vertebrate Animals)

Biohazards:

Not Applicable (No Biohazards).

Resource Sharing Plans:

Acceptable.

Authentication of Key Biological and/or Chemical Resources:

Not Applicable (No Relevant Resources)

Budget and Period of Support:

Recommend as Requested.

CRITIQUE 2

Significance: 2
Investigator(s): 1
Innovation: 1
Approach: 2
Environment: 1

Overall Impact: This new application from an early-stage investigator proposes to conduct a large (N=988), randomized trial of nipple stimulation using breast pumps for labor induction. The investigators will conduct the trial at 2 academic medical center sites, randomizing patients undergoing

induction of labor to nipple stimulation for at least 2 hours versus immediate synthetic oxytocin without nipple stimulation. In Aim 1 the investigators will examine the effect of the intervention on the rate of spontaneous vaginal delivery, as well as other labor complications. In Aim 2 the investigators will examine the effect of the intervention on lactation outcomes (primary: exclusive breast milk at hospital discharge). In Aim 3 they will conduct a cost-effectiveness analysis. Strengths of the application include the huge potential public health impact given that labor induction is incredibly common and costly. A low-cost intervention that could lower the cesarean delivery rate or improve breastfeeding rates could lead to vast improvement in maternal and neonatal morbidity. The biological rationale supporting the research is strong and there is compelling pilot data (a pilot RCT with n=100) supporting feasibility. The multicenter randomized control trial design is another major strength. Minor limitations include that adherence to the protocol would likely be enhanced if formal nursing and/or lactation support for the intervention was built into the enrollment process and if more research staff time allocation was planned at the 2nd study site. The variability in discharge timing may introduce bias into the assessment of Aim 2 outcomes. There is a possibility that there will be substantial attrition for the surveys in the postpartum period, but the research plan mitigates this by focusing on outcomes that will be assessed either at hospital discharge or in the short term. Finally, it would be difficult to conduct a double-blind trial of this intervention, which is a limitation, but is understandable.

1. Significance:

Strengths

- Labor induction is an extremely common procedure used for a variety of indications with significant limitations: requires oversight/monitoring, increased risk of operative delivery when oxytocin use is prolonged, does not penetrate blood-brain barrier. Costs associated with labor induction are significant.
- There is a strong scientific rationale, as breast nipple stimulation leads to pulsatile release of endogenous oxytocin mimicking spontaneous contractions and can cause uterine contractions.
- The preliminary data from the pilot RCT is compelling as there was a higher vaginal delivery rate (Vaginal delivery in 73% of nipple stimulation vs 63% in control group, non-significant), shorter length of induction, and lower oxytocin dose required in the intervention group. There were also subjectively assessed maternal breastfeeding outcomes.

Weaknesses

- None noted.

2. Investigator(s):

Strengths

- The PI Dr. Son is an obstetric clinical trialist with expertise in labor management and intrapartum oxytocin. Impressive track record for early career stage including a prior large RCT.
- Co-I Dr. Stetson is the medical director of L&D at Northwestern. Site PI at NW. She has previously collaborated extensively with Dr. Son.
- Co-I Dr. Taylor is a neonatologist and expert in infant nutrition and growth and breastfeeding.
- Co-I Dr. Shabanova is a biostatistician with expertise in perinatal research.
- Co-I Dr. Xu is a health economist who will conduct the cost-effective analysis for Aim 3.

Weaknesses

- None noted.

3. Innovation:

Strengths

- The proposal was considered highly innovative in that it uses a low cost intervention that has biologic plausibility and is paradigm shifting in terms of methods of labor induction.
- The inclusion of both labor outcomes and breastfeeding outcomes is innovative.

Weaknesses

- None noted.

4. Approach:

Strengths

- The primary and secondary outcomes are important, have a biological basis, are feasible to assess, and are well-reasoned.
- Many aspects of the approach have been informed by a pilot trial of n=100 at Yale, providing effect size estimates and supporting feasibility. This showed that 44% of approached patients consented to the intervention and enrolled and that nulliparas were equally likely to consent as multiparas. The intervention was well-tolerated, and the median duration of nipple stimulation was 3.5 hours.
- Inclusion of a cost-effectiveness analysis is a strength.
- Multicenter trial.
- Intention to treat analysis with block randomization by study site and amniotic membrane status.
- There are many letters of support provided from clinical groups who deliver at the study site hospitals. It is critically important to have the buy-in of the obstetric providers.
- The power calculations are based on solid preliminary data and are well-executed.
- Uterine tachysystole was addressed in the safety protocol for the trial.

Weaknesses

- The trial will not be blinded, which is reasonable given the intervention. Although the main outcomes are objective measures, it would be good to blind the outcome assessors to the intervention if possible.
- Because hospital discharge occurs at different time points, there will be a lack of standardization of the time certain outcomes are assessed, including the main outcome for Aim 2 (exclusive breastfeeding at discharge) and the Aim 2 secondary outcome maximum percent weight loss. This assessment could be biased, particularly if there is a differential vaginal vs cesarean delivery rate in the 2 groups. The investigators recognize this and will attempt to adjust for this statistically, but likely statistical adjustment will not complete eliminate this issue.
- For Aim 3, many self-reported measures need to be collected from participants and there may be substantial attrition in the postpartum period, especially at the 6 month time point. However, the primary analysis only includes up to 2 weeks postpartum which is a reasonable compromise.

- Adherence to the protocol would likely be enhanced if formal nursing and/or lactation support for the intervention was built into the enrollment process, particularly because the study involves nulliparous participants who may not have experience with breast pumps.
- The research staff allocation at Northwestern may be inadequate for the number of participants to be enrolled there.

5. Environment:

Strengths

- The two sites for the trial are high-performing academic medical centers with a record of success in completing trials and clinical studies in obstetrics.
- Yale New Haven Hospital.
- Northwestern Memorial Hospital.
- Strong support from clinical leadership and involved clinical providers (including lactation, nurse mid-wives, private practices) at both sites which will be essential to completing the trial.

Weaknesses

- None noted.

Study Timeline:

Strengths

- Study start up time is appropriate and participant accrual plans are reasonable.
- The milestones are clear.
- There is sufficient time at the end of the grant in case the participant accrual is delayed

Weaknesses

- None noted.

Protections for Human Subjects:

Acceptable Risks and/or Adequate Protections

- The protections are acceptable. However, the incentives are a bit low for the amount of participant time that this will require.

Data and Safety Monitoring Plan (Applicable for Clinical Trials Only):

- Not applicable

Inclusion Plans:

- Sex/Gender: Distribution justified scientifically
- Race/Ethnicity: Distribution justified scientifically
- For NIH-Defined Phase III trials, Plans for valid design and analysis: Scientifically acceptable
- Inclusion/Exclusion Based on Age: Distribution justified scientifically

Vertebrate Animals:

Not Applicable (No Vertebrate Animals)

Biohazards:

Not Applicable (No Biohazards).

Resource Sharing Plans:

Acceptable.

Authentication of Key Biological and/or Chemical Resources:

Not Applicable (No Relevant Resources)

Budget and Period of Support:

Budget Modifications Recommended (in amount/time)

Recommended budget modifications or possible overlap identified:

- The clinical research staff at Northwestern appear under supported given that they are going to enroll half of the participants. Recommend increasing the budget at Northwestern.

CRITIQUE 3

Significance: 4
Investigator(s): 3
Innovation: 4
Approach: 4
Environment: 3

Overall Impact: This new submission by a new early-stage investigator is a RCT of 988 nulliparous women to determine if nipple stimulation via electric breast pump for induction increases SVD and breastfeeding. PI has experience with clinical trials in that she recently completed a large trial comparing high and low dose oxytocin for augmentation of labor. They have data from a pilot study of 100 randomized women. Even if this trial shows that using a breast pump during labor decreases the time to delivery and increases breastfeeding it may be difficult to get women to wear this for such a long time. Only 44% of women approached in their pilot study agreed to participate. Using nipple stimulation with a breast pump during labor will prevent the immediate control of the oxytocin infusion which the provider has with IV infusion which could lead to more uterine tachysystole placing the fetus at increased risk for intrapartum hypoxia-ischemia. It is unknown how prolonged breast pump use will correlate with oxytocin levels. Short term use similar to the duration of average human breastfeeding may be pulsatile, but prolonged use during an entire labor could lead to increased levels which could increase the risk of tachysystole or downregulate receptors leading to decreased levels that wouldn’t be effective in decreasing the duration of labor.

1. Significance:

Strengths

- Address a significant clinical problem in that > 1 million women are induced each year, and oxytocin infusions are a source of medicolegal controversy related to safety concerns. If nipple stimulation during labor with a breast pump increases the rate of vaginal deliveries and breast feeding this would offer significant health advantages to mother and baby in addition to cost savings.

Weaknesses

- Many women already complain about being tethered to an electronic fetal monitor during labor, and it may be difficult to get them to wear a breast pump during labor for such a long period of time. Only 44% of the women approached for their pilot study agreed to participate.
- Uterine tachysystole is a concern during labor, and IV oxytocin protocols are designed to specifically avoid it. When it occurs it puts the fetus at risk which can be mitigated by immediately stopping the IV oxytocin. With nipple stimulation via a breast pump the provider won't have control of the oxytocin dose and won't be able to immediately stop the oxytocin. This could mean that the fetus is exposed to tachysystole and resultant hypoxia-ischemia longer.

Study Timeline:

Strengths

- Appropriately designed RCT with planned enrollment of 988 nulliparas at 2 university hospitals with annual delivery volumes of 5,700 and 12,000. They will act as the single IRB.

Weaknesses

- None noted.

Protections for Human Subjects:

Acceptable Risks and/or Adequate Protections

- The proposed study has greater than minimal risk because the data regarding the use of intrapartum nipple stimulation therapy via electric breast pump (i.e., study intervention) remain limited. However, risks are not anticipated to be high given the reassuring data from the pilot study of 100 women, the existing literature, and because this therapy is thought to be more physiological than synthetic oxytocin.

Data and Safety Monitoring Plan (Applicable for Clinical Trials Only):

Acceptable

- They will establish an independent study-specific Data Safety and Monitoring Board (DSMB) to oversee the trial. The DSMB will consist of 4 members who are completely independent of the trial and have no financial, scientific, or other conflicts of interest with the trial.

Inclusion Plans:

- Sex/Gender: Distribution justified scientifically
- Race/Ethnicity: Distribution justified scientifically
- For NIH-Defined Phase III trials, Plans for valid design and analysis: Not applicable
- Inclusion/Exclusion Based on Age: Distribution justified scientifically

- Recruitment plan, as well as inclusion and exclusion criteria, are well described and appropriate.

Vertebrate Animals:
Not Applicable (No Vertebrate Animals)

Biohazards:
Not Applicable (No Biohazards)

Resource Sharing Plans:
Acceptable.

Authentication of Key Biological and/or Chemical Resources:
Not Applicable (No Relevant Resources)

Budget and Period of Support:
Recommend as Requested.

THE FOLLOWING SECTIONS WERE PREPARED BY THE SCIENTIFIC REVIEW OFFICER TO SUMMARIZE THE OUTCOME OF DISCUSSIONS OF THE REVIEW COMMITTEE, OR REVIEWERS' WRITTEN CRITIQUES, ON THE FOLLOWING ISSUES:

PROTECTION OF HUMAN SUBJECTS: ACCEPTABLE

INCLUSION OF WOMEN PLAN: ACCEPTABLE

INCLUSION OF MINORITIES PLAN: ACCEPTABLE

INCLUSION ACROSS THE LIFESPAN: ACCEPTABLE

COMMITTEE BUDGET RECOMMENDATIONS: The budget was recommended as requested.

Footnotes for 1 R01 HD111633-01; PI Name: Son, Moeun

NIH has modified its policy regarding the receipt of resubmissions (amended applications).See Guide Notice NOT-OD-18-197 at https://grants.nih.gov/grants/guide/notice-files/NOT-OD-18-197.html. The impact/priority score is calculated after discussion of an application by averaging the overall scores (1-9) given by all voting reviewers on the committee and multiplying by 10. The criterion scores are submitted prior to the meeting by the individual reviewers assigned to an application, and are not discussed specifically at the review meeting or calculated into the overall impact score. Some applications also receive a percentile ranking. For details on the review process, see http://grants.nih.gov/grants/peer_review_process.htm#scoring.
